# Light and Pollination Limitation Alter Patterns of Fitness and Phenotypic Selection in *Sagittaria trifolia* L.: Insights From Sequential Inflorescences

**DOI:** 10.1002/ece3.73955

**Published:** 2026-07-01

**Authors:** Hanqing Tang, Can Dai

**Affiliations:** ^1^ School of Resources and Environmental Science Hubei University Wuhan China; ^2^ Hubei Key Laboratory of Regional Development and Environmental Response Hubei University Wuhan China

**Keywords:** female reproductive success, phenotypic selection, pollinator limitation, reproductive opportunities, resource reallocation

## Abstract

Seed production in plants is constrained by both light and pollination. Studies often focus on the effect of each factor separately on subsets of plants, leaving the combined constraints of light and pollination on individual fitness poorly understood. Here, we placed arrays of the monoecious herb *Sagittaria trifolia* L. in three distinct treatments, namely, a “mesh‐enclosed” treatment where plants were covered with a fine mesh, simultaneously restricting light availability and pollinator access, a “shaded” treatment with limited light due to canopy cover, and an “open” treatment with abundant sunlight. Flowering traits and reproductive fitness were measured at both the inflorescence and individual levels. Plants in the open treatment produced the most inflorescences, flowers, and seeds. While initial fruit‐set was the lowest in the mesh‐enclosed treatment, it increased markedly in later inflorescences. Despite severe pollinator limitation, mesh‐enclosed plants offset early fitness costs by producing more inflorescences, ultimately achieving total seed output similar to shaded plants. Phenotypic selection on five measured traits was stronger under limited conditions directed toward more female flowers per inflorescence in shade, but toward more inflorescences in mesh‐enclosed plants. The results suggest that the effect of pollinator limitation in *S. trifolia* could be mitigated by the allocation of more inflorescences. Our study emphasizes the importance of considering temporal dynamics across sequential inflorescences within a single flowering season for accurately quantifying phenotypic selection and whole‐plant reproductive strategies.

## Graphical abstract

We examined how light limitation and reduced pollinator access influence female fitness and phenotypic selection in *Sagittaria trifolia* across sequential inflorescences. Open‐grown plants produced the most inflorescences, flowers, and seeds, whereas mesh‐enclosed plants compensated for low early fruit set through later reproductive adjustment and ultimately matched shaded plants in seed output. Our results highlight the importance of temporal reproductive dynamics in buffering pollination limitation under resource‐constrained conditions.

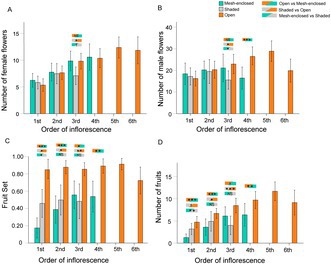

## 1 Introduction

Plant reproductive success is constrained by both resource availability and pollination service. Female reproduction, in particular, demands substantial resources for seed and fruit development (Williams 1966; Obeso 2002). However, pollen limitation, which reduces fruit and seed production relative to the maximum potential under unlimited pollen receipt—is increasingly recognized as a widespread phenomenon in both wild and cultivated plants (Rodger et al. 2021; Turo et al. 2024). Rather than viewing these as independent constraints, a more integrated framework suggests that fruit production is often simultaneously limited by both pollen and resources (Haig and Westoby 1988; Brookes et al. 2008), and that the relative importance of each can vary across environmental contexts (Ashman et al. 2004).

Critically, plant resource budgets are dynamic over the growing season. Plants balance continuous growth, flowering, and fruiting against photosynthetic gains, leading to dynamic shifts in resource allocation (Burd 2016). Moreover, due to the modular construction, plants often exhibit considerable temporal variation in reproductive investment across successive flowering events (Wesselingh 2007). Lloyd (1980) proposed the hypothesis of serial adjustment, which posits that in species with extended or sequential flowering, reproductive allocation is not fixed at the outset of the season; instead, plants may adjust their allocation strategy throughout the reproductive period based on resource availability and environmental feedback. However, many empirical studies focus on only a subset of flowers or a single inflorescence per plant (Wang et al. 2023; Hou et al. 2024), thereby overlooking potential trade‐offs and reallocation strategies across the entire reproductive season. Such a limited perspective may ignore whole‐plant responses to stress, such as adjustments in flowering pace, flower number, or sequential investment in later inflorescences (Obeso 2002; Ashman et al. 2004; Schwarz et al. 2020). Furthermore, cumulative seed production, rather than estimates extrapolated from a fraction of a season, should be more indicative of individual fitness. An accurate assessment of reproductive fitness is especially relevant when studying phenotypic selection, as both the opportunity for selection (*I*) and trait‐fitness covariance rely heavily on the distribution of fitness in a population (Lande and Arnold 1983; Arnold and Wade 1984; Tonnabel et al. 2019).

Pollen limitation appears to be widespread worldwide (Archer et al. 2014; Rodger et al. 2021), but understanding its ecological and evolutionary consequences requires not only testing whether reproduction is pollen‐limited, but also examining how plants respond when pollinator availability is reduced. Reducing pollinator access using mesh enclosures provides one way to address this latter question (e.g., Brown and Caruso 2023), because such enclosures can mimic reduced visitation by major flying pollinators expected under global declines in key pollinator taxa, such as honey bees, bumble bees, solitary bees, hoverflies, and butterflies (Potts et al. 2010). This approach may also allow assessment of compensatory ecological processes, such as pollination by alternative, often less‐efficient visitors that can enter or remain active within enclosed environments (Gómez et al. 1996; Jaeger et al. 2023). Empirical evidence indicates that when dominant pollinators decline, visitation by alternative taxa may increase and partially compensate for pollination deficits (Rader et al. 2016).

The interplay between resources and pollination likely influences not only mean fitness but also the strength and direction of phenotypic selection on reproductive traits (Sletvold et al. 2017; Wu et al. 2023). It remains poorly understood how combined stress from pollen and resource limitation alters selection on flower traits, such as floral display that influences pollinator attraction, or on whole‐plant strategies, such as reproductive duration and inflorescence number (Burd 2008; Rosenheim et al. 2014). Only a handful of studies have examined phenotypic selection under concurrent resource and pollen limitation. Sletvold et al. (2017) reported weak resource effects on fitness and selection intensity on floral display traits in the orchid *Dactylorhiza lapponica*, whereas Wu et al. (2022, 2023) found that soil water and nutrient availability interactively modified pollinator‐mediated directional and correlational selection on floral display (flower number and size) in *Primula tibetica*, with outcomes that varied across traits and environmental contexts.

In this study, we investigate how variation in resource availability and pollination service is associated with flowering and reproduction across multiple inflorescences of the monoecious herb *Sagittaria trifolia* in a reproductive season. Clonally propagated *S. trifolia* were arranged in potted arrays across three environmentally distinct treatments (Figure S1): a “mesh‐enclosed” site under reduced light, in which plants were covered with netting to restrict access of major flying pollinators, a “shaded” site with reduced light availability (lower resources) and natural pollinator visitation (Luo et al. 2018), and an “open” site with full light (high resources) and natural pollinator visitation (Tang et al. 2023). Thus, the “mesh‐enclosed” treatment represents a scenario of resource limitation combined with restricted access to effective pollinators, rather than pollinator exclusion. We quantify not only reproductive traits for each inflorescence but also plant‐level performance traits related to vegetative size and storage investment (plant height and corm number), and we evaluate phenotypic selection using whole‐season female fitness (total seed production). We address the following questions: (1) How do environmental contexts affect overall female reproductive success (total seed production) in *S. trifolia* ? (2) Does the temporal pattern in the production of flowers and fruits across sequential inflorescences vary among the three treatments? (3) How does phenotypic selection on measured reproductive traits vary among the three treatments?

## 2 Materials and Methods

*Sagittaria trifolia* , commonly known as arrowhead, is a monoecious herb belonging to the Alismataceae family. It thrives in open shallow‐water areas such as ponds, marshes, and paddy fields, and can also be found in understory ditches and lakesides, exhibiting wide distribution throughout Asia. Being self‐compatible, the plant can set fruits through both self‐ and cross‐pollination, with an outcrossing rate of approximately 80% under open‐pollinated conditions (Dai, Li, et al. 2018). Because male and female flowers are spatially separated within each inflorescence, autonomous self‐pollination is unlikely; pollinator visitation is required for pollen transfer, even for self‐pollen. Multiple inflorescences bloom successively during a single reproductive season, with flowers on each inflorescence opening from bottom to top and female flowers opening first. Individual flowers are ephemeral, lasting approximately 1 day, and multiple flowers may open simultaneously within an inflorescence. The plants die back in winter, and rootstocks rarely survive from year to year. The corm serves as the mode of vegetative reproduction for *S. trifolia*, and a plant usually produces several corms each year. This study involved the collection of nearly 100 *S. trifolia* plants from 22 natural populations in Hubei Province of China (referring to the site map in Zhou et al. 2020). To ensure genetic diversity, samples were collected at a minimum distance of 10 m between plants in each population. The plants were kept in a nursery house and propagated through corms in the Wuhan Botanical Garden of the Chinese Academy of Sciences (WBG; 114.42° E, 30.54° N). At the beginning of the experiment in year 2014, the corm offspring were cultivated in three distinct treatments at WBG.

The experimental environments consisted of (i) a mesh‐enclosed treatment in which plants were covered with netting to restrict access by major flying pollinators, (ii) a shaded treatment bordered by tall trees that provided an understory habitat, and (iii) an open treatment with full sun access (Figure S1 and Figure S2). The mesh‐enclosed treatment was intended to restrict access of larger flying visitors; however, small or crawling insects could still enter, so this treatment represents restricted visitation rather than complete pollinator exclusion. Because pollinator restriction was implemented only under reduced light, our design does not fully isolate resource availability and pollination service and does not allow a formal test of their interaction; instead, we used these habitat contrasts to evaluate how combined constraints shape overall reproduction and allocation across sequential inflorescences.

For each *S. trifolia* genotype, three corms of similar size were selected, transplanted into pots, and randomly assigned to the three treatments on 27 April, ensuring a uniform initial stage and similar genetic composition across treatments. Additionally, some extra corms were planted to provide supplements in case of death during transplant shock and early growth. At the sprouting stage, the sample sizes of three treatments were finalized as 52 in the mesh‐enclosed treatment, 49 in the shaded treatment and 50 in the open treatment.

Light intensity (PAR, μmol·m^−2^·s^−1^) was measured using quantum meters (MQ‐200, Apogee, Utah, USA) at 08:00, 10:00, 12:00, 14:00, 16:00 and 18:00 for three consecutive days at random positions within each treatment. Based on field observations (Figure S2), the main pollinators in the open treatment were honeybees (*Apis* spp.), whereas hoverflies (Syrphidae) dominated visits in the shaded treatment; in the mesh‐enclosed treatment, floral visitors were largely limited to crawling insects (e.g., ants *Camponotus* spp. and true bugs *Eysarcoris* spp.). Please see Luo et al. (2018) for detailed comparisons of pollinator composition and visitation behavior between the open and shaded habitats.

All plants in three treatments were allowed to flower and fruit naturally throughout the reproductive season from mid‐July to late‐September. The proportion of reproduction was defined as the proportion of plants in each treatment that survived and flowered, and the date of first flower was a record of the number of days after transplant (27th April) when the first flower opened in a plant. Subsequently, the flowering status of each plant in the three treatments was documented daily, that is, the number of female and male flowers blooming per day per inflorescence. The percentage of female flowers was calculated based on the number of female and male flowers tallied in all inflorescences of a plant. The duration of anthesis was manifested by the number of days from the first to the last flower of each plant. The total number of inflorescences produced by each plant during the entire flowering season was recorded. At the end of the season, the number of corms per plant was counted as a measure of vegetative reproductive investment. We measured the height of each plant for the tallest leaf from the soil‐stem junction to the length of the petiole in the middle of the anthesis (5th September). To prevent mature fruits from shedding seeds, fruits were collected every week during and after anthesis, extending up to 6th November when most plants began senescence. The number of fruits and fruit‐set were then recorded for each inflorescence. During the entire cultivation period, all plants in each treatment underwent consistent periodic fertilization, with pest control measures maintained. In case that plants died naturally after the onset of experimental records, no intervention was performed and thus the number of plants survived in three treatments finally varied (mesh‐enclosed: 35, shaded: 34, open: 43).

Upon the collection of fruits, we randomly chose three fruits from each inflorescence (all if less than three) and carried out seed counting in the laboratory following the methodology in Dai, Luo, et al. (2018). Based on the seed count per fruit sample, we calculated the number of seeds, which serves as the indicator of the female fitness of a plant, by summing the seed number in all inflorescences, each of which was a product of the average seed count and the fruit number of a particular inflorescence.

### 2.1 Statistical Analysis

Statistical analyses were performed using *R* version 4.0.4 (R Core Team 2021). Data manipulation was carried out with *dplyr* (Wickham et al. 2023) and *tidyr* (Wickham et al. 2024). To examine differences in the growth and reproduction of *Sagittaria trifolia* among treatments, we first compared the proportion of reproductive plants across the three treatments using a chi‐squared test of independence. Treatment was treated as a three‐level factor (mesh‐enclosed, shaded, and open).

At the individual level, we analyzed plant height, date of first flower, duration of anthesis, number of inflorescences, average numbers of female and male flowers per inflorescence, percentage of female flowers, seed production, and corm number. A preliminary model‐screening step was used to evaluate candidate transformations and assess whether variables were more appropriately analyzed under Gaussian or non‐Gaussian mixed‐effects models. Final models were then fitted according to the distributional properties of each response variable, with treatment as a fixed effect and genotype as a random intercept. Plant height, date of first flower, duration of anthesis, and seed production were analyzed using linear mixed‐effects models (LMMs) fitted with *lme4* (Bates et al. 2015) and *lmerTest* (Kuznetsova et al. 2017), with date of first flower and seed production square‐root transformed. Count traits, including corm number, number of inflorescences, and the average numbers of female and male flowers per inflorescence, were analyzed using generalized linear mixed‐effects models (GLMMs) fitted with *glmmTMB* (Brooks et al. 2017) with a Poisson error distribution, and negative binomial models were considered when overdispersion was detected. Percentage of female flowers was analyzed using a beta‐binomial mixed‐effects model fitted with *glmmTMB* based on the numbers of female and male flowers, with treatment as a fixed effect and genotype as a random intercept. Pairwise comparisons among treatments were conducted using estimated marginal means (*emmeans*; Lenth 2022) with Tukey adjustment via *multcomp* (Hothorn et al. 2008), and results are reported as estimated marginal means ± s.e. Plants that survived to reproduction but produced no fruits or seeds were retained in analyses with fitness = 0, as excluding them would truncate the fitness distribution and bias selection estimates upward. Individuals that died before or during the reproductive period were excluded from selection analyses, as their failure to reproduce could not be attributed to the measured traits.

At the inflorescence level, inflorescences were assigned an order based on their blooming sequence within each plant (1st, 2nd, 3rd, etc.). Because higher‐order inflorescences were produced by relatively few plants in some treatments, sample sizes became sparse and uneven across inflorescence orders. To reduce bias associated with small and unbalanced sample sizes, direct comparisons among all three treatments were restricted to inflorescence orders 1–3. In addition, because the fourth inflorescence order was sufficiently represented only in the mesh‐enclosed and open treatments, comparisons for order 4 were performed only between these two treatments. For each eligible inflorescence order, the numbers of female flowers, male flowers, and fruits were analyzed using GLMMs fitted with *glmmTMB* with a Poisson error distribution and genotype as a random intercept; negative binomial models were considered if overdispersion was detected. Fruit set, given detected overdispersion, was analyzed using a beta‐binomial mixed‐effects model fitted with *glmmTMB* with genotype as a random intercept. Inflorescences with zero female flowers were excluded from fruit‐set analyses. Pairwise comparisons among treatments were conducted using estimated marginal means with Tukey adjustment.

To evaluate whether flowering and fruiting traits changed across successive inflorescences and whether these trajectories differed among treatments, we fitted repeated‐measures mixed‐effects models, with repeated observations from successive inflorescences of the same plant tracked using individual plant identity as a grouping factor. Genotype was included as a separate random effect to account for genetic relatedness among clonal replicates across treatments. Treatment, inflorescence order, and their interaction were included as fixed effects, with inflorescence order treated as a continuous covariate. For female flowers, male flowers, and fruits, genotype was included as a random intercept, and repeated observations within plants were modeled using a first‐order autoregressive correlation structure. Fruit set was analyzed using a beta‐binomial mixed‐effects model, with the numbers of fruits and non‐fruiting female flowers specified as the binomial response. The initial fruit‐set model also included genotype as a random intercept, but this term was removed because the full model was not estimable. Fixed effects were tested using likelihood‐ratio tests based on nested model comparisons.

Light intensity was analyzed using a linear mixed‐effects model, with treatment as a fixed effect and time point as a random factor. For Gaussian models, model assumptions were evaluated using residual diagnostics, including Q–Q plots and residual‐versus‐fitted plots. Homogeneity of variances was assessed using Levene's tests (*car*; Fox and Weisberg 2019). For Poisson and binomial distributed data, overdispersion was assessed using Pearson residual‐based dispersion statistics.

Correlation matrices were constructed using *psych* (Revelle 2024) and *Hmisc* (Harrell Jr 2022) to show the Pearson's correlation coefficients among the traits of the plants in each treatment. Selection opportunity in each treatment was quantified as the variance in relative female fitness, where relative female fitness was calculated as the total number of seeds produced by an individual divided by the mean seed production of all plants in that treatment. Differences in selection opportunity among treatments were assessed using Levene's test. Correlation matrices were constructed for descriptive purposes using *psych* (Revelle 2024) and *Hmisc* (Harrell Jr 2022); *p*‐values are reported without correction for multiple testing and should be interpreted as exploratory. Because the phenotypic variation used to estimate selection gradients was derived from multiple source populations distributed across treatments, this broader range facilitates the detection power for processes and targeted traits of natural selection, but it may also inflate the opportunity for selection relative to single‐population estimates.

We estimated linear selection gradients (*β*) using multiple regression, with all five traits entered simultaneously in a single model, following the framework of Lande and Arnold (1983). Relative female fitness was regressed on phenotypic traits, including plant height, duration of anthesis, number of inflorescences, and the mean numbers of female and male flowers per inflorescence. The percentage of female flowers was originally in the model but finally excluded due to its high correlation with the number of female and male flowers per inflorescence (Table S1). The variance inflation factor (VIF) for the remaining trait variables was < 5, suggesting no serious multicollinearity (Menard 2002). We chose these traits to capture resource acquisition (plant height, corm number) and reproductive deployment at inflorescence and whole‐plant levels (flower numbers, inflorescence number, flowering duration). Phenotypic traits were standardized by centering each individual's trait value around the treatment mean and scaling by the trait's standard deviation, thus achieving a standardized mean of 0 and standard deviation of 1. The 95% bias‐corrected confidence intervals for the selection gradients and *p*‐values were determined using parametric bootstrapping with 10,000 resamplings (Hou et al. 2024). To test whether selection gradients differed among treatments, we fitted ANCOVA models with relative female fitness as the response and treatment, each standardized trait, and their interaction as fixed effects. A significant trait × treatment interaction indicates that the selection gradient on that trait varies among treatments.

## 3 Results

### 3.1 Treatment Effects on Traits and Fitness

**FIGURE 1:**
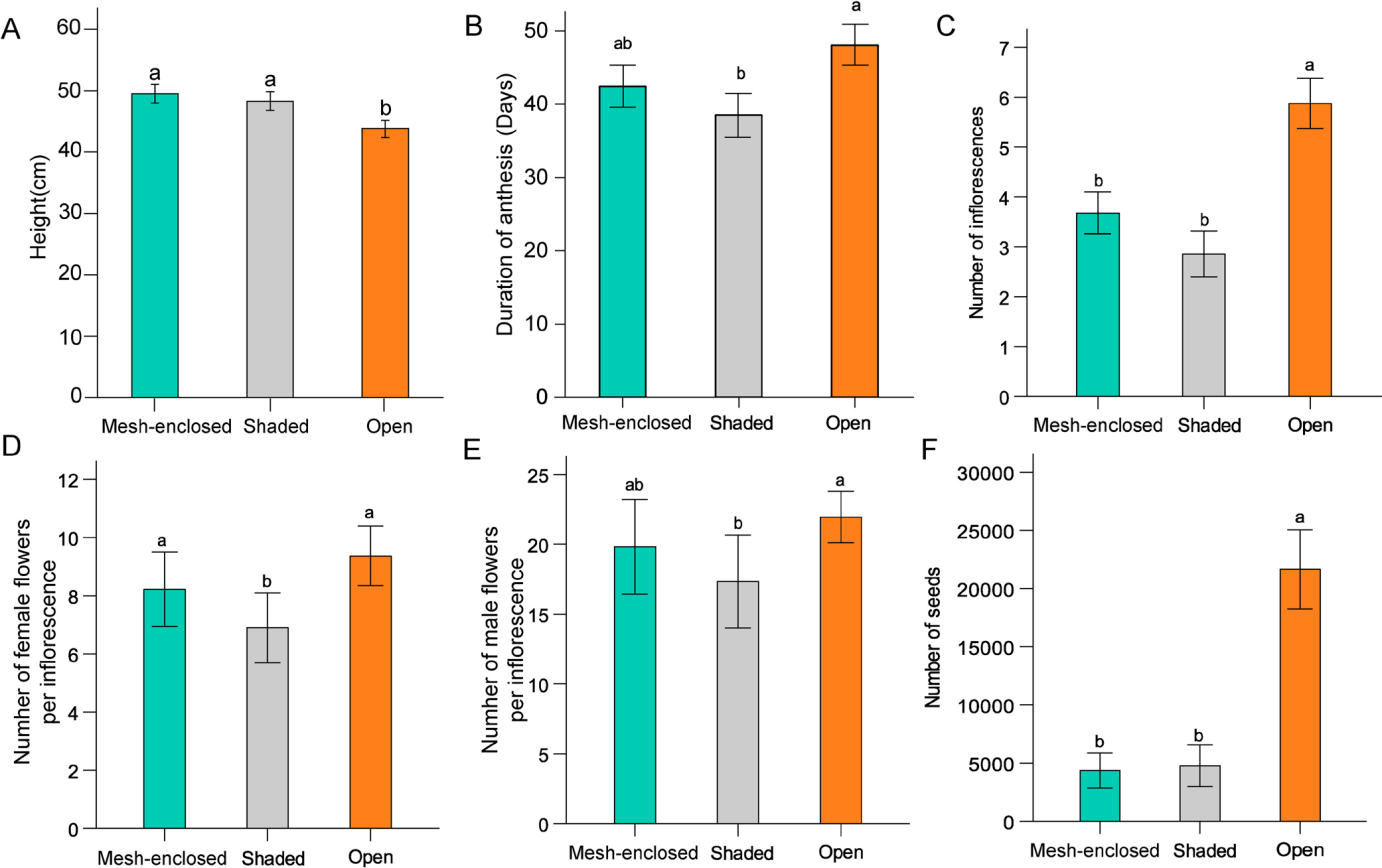
Comparisons of reproductive traits among plants of *Sagittaria trifolia* growing in the three treatments (mesh‐enclosed in turquoise, shaded in gray and open in orange): Plant height (A), duration of anthesis (B), number of inflorescences (C), number of female flowers per inflorescence (D), number of male flowers per inflorescence (E), and number of seeds (F). Columns share the same lowercase letters are not significantly different at the level of α = *0.05*.

Light intensity in the mesh‐enclosed treatment did not differ significantly from that in the shaded treatment (Figure S2), confirming that both experienced similarly reduced light relative to the open treatment. In the mesh‐enclosed and shaded treatments where light intensity was lower, the proportions of plants that survived and entered reproduction were 67.3% and 69.4%, respectively, which were notably lower than the 86% observed in the open treatment (mesh‐enclosed vs. shaded *X* ^2^ = 0, *p* = 0.99; mesh‐enclosed vs. open *X* ^2^ = 4.0, *p* = 0.046; shaded vs. open *X* ^2^ = 3.0, *p* = 0.08). Furthermore, plants in the mesh‐enclosed and shaded treatments exhibited significantly larger plant height than those in the open treatment (*F*_2,69.5_ = 7.97, *p* = 0.0008; Figure 1A).

Regarding reproductive features, there was no difference among three treatments in the date of first flower (*F*_2,73.8_ = 0.74, *p* = 0.48; mesh‐enclosed: 67.2 ± 2.5 days, shaded: 65.9 ± 2.6 days, open: 62.9 ± 2.4 days). However, most other traits exhibited significant differences among the treatments. The duration of anthesis was significantly shorter in the shaded treatment compared to the open, with plants in the mesh‐enclosed treatment showing intermediate values (*F*_2,106_ = 3.04, *p* = 0.05; Figure 1B). The number of inflorescences per plant was significantly higher in the open treatment than in the mesh‐enclosed and shaded treatments, whereas the latter two did not differ significantly (*χ* ^*2*^ = 36.5, *p* < 0.0001; Figure 1C). The number of female flowers per inflorescence was significantly reduced in the shaded treatment relative to the other two (*χ* ^*2*^ = 14.54, *p* = 0.0007; Figure 1D). The pattern of male flowers was comparable to female ones (*χ* ^*2*^ = 8.40, *p* = 0.015; Figure 1E). In terms of sex allocation, there were no significant differences among the treatments in the proportion of female flowers (*χ* ^*2*^ = 0.59, *p* = 0.75; mesh‐enclosed: 0.31 ± 0.01, shaded: 0.30 ± 0.02, open: 0.30 ± 0.01). Seed yield differed significantly among treatments (*F*_2,72.4_ = 74.8, *p* < 0.0001; Figure 1F): seed production in the open treatment was significantly higher than in both the mesh‐enclosed and shaded treatments, whereas the mesh‐enclosed and shaded treatments did not differ. Furthermore, plants in the mesh‐enclosed, shaded and open treatments produced 9.11 ± 1.01, 4.71 ± 1.02 and 13.12 ± 0.94 corms, respectively, and corm number differed significantly among the three treatments (*χ* ^*2*^ = 33.88, *p* < 0.0001; mesh‐enclosed vs. shaded, *p* = 0.00022; mesh‐enclosed vs. open, *p* = 0.037; shaded vs. open, *p* < 0.0001).

### 3.2 Floral Allocation Across Sequential Inflorescences

**FIGURE 2:**
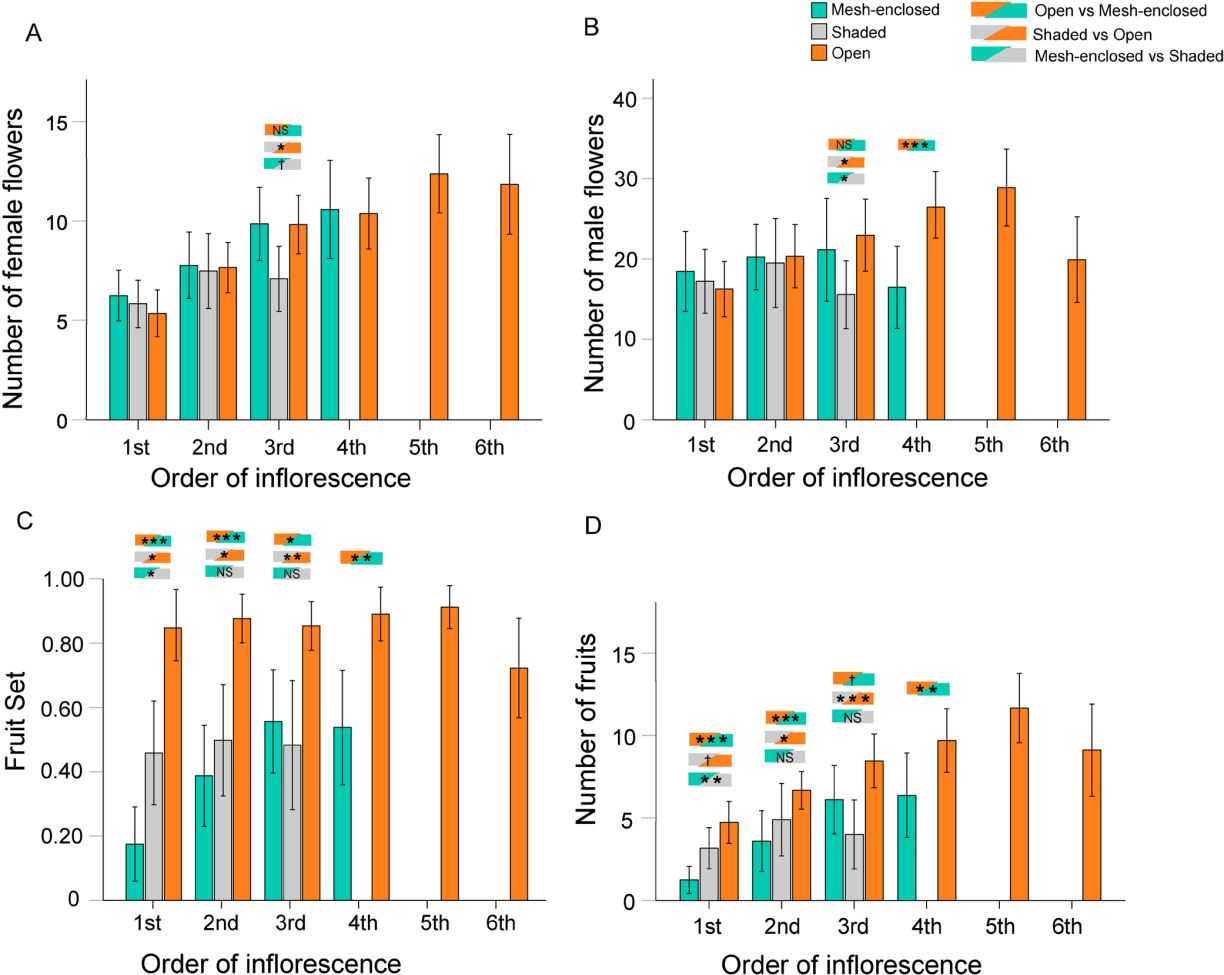
Number of female flowers (A), number of male flowers (B), fruit set (C), and number of fruits (D) in sequential inflorescences of *Sagittaria trifolia* growing in three treatments (mesh‐enclosed in turquoise, shaded in gray, and open in orange). NS indicates *p* > 0.1, †indicates *p* < 0.1, * *p* indicates < 0.05, ** indicates *p* < 0.01 and *** indicates *p* < 0.0001.

When flower production was examined at the inflorescence level, differences among treatments were mainly expressed at the 3rd inflorescence. For female flowers, plants in the shaded treatment produced fewer flowers than those in the open and mesh‐enclosed treatment at the 3rd inflorescence (Figure 2A; Table 1). For male flowers, the shaded treatment also showed reduced flower production at the 3rd inflorescence (Figure 2B; Table 1). After the 3rd inflorescence, plants in the shaded treatment had nearly completed flowering, whereas plants in the mesh‐enclosed and open treatments continued to produce additional inflorescences. Meanwhile, the numbers of female flowers increased with inflorescence order in the mesh‐enclosed and open treatments, but not in the shaded treatment (Table S2).

**TABLE 1:** Results of mixed‐effect models testing environmental effects on inflorescence‐level reproductive traits (female flowers, male flowers, fruit‐set, and fruit number) of *Sagittaria trifolia* . Bold *p*‐values indicate statistical significance (*p* < 0.05).

Trait	Inflorescence	df	*χ* ^*2*^	*p*
Female flowers	1st	2	1.56	0.46
	2nd	2	0.08	0.96
	3rd	2	6.26	**0.04**
	4th	1	0.002	0.96
Male flowers	1st	2	0.20	0.90
	2nd	2	0.60	0.74
	3rd	2	9.48	**0.009**
	4th	1	10.57	**0.001**
Fruit‐set	1st	2	49.91	**< 0.0001**
	2nd	2	20.09	**< 0.0001**
	3rd	2	15.71	**0.0004**
	4th	1	13.05	**0.0003**
Number of fruits	1st	2	29.2	**< 0.0001**
	2nd	2	17.74	**0.0001**
	3rd	2	16.04	**0.0003**
	4th	1	6.89	**0.009**

For fruit‐set, plants in the open treatment consistently showed the highest fruit‐set across sequential inflorescences (Figure 2C; Table 1). Plants in the mesh‐enclosed treatment had a substantially lower fruit‐set than those in the shaded treatment at the 1st inflorescence, but this difference gradually diminished in later inflorescences (Figure 2C, Table 1). This pattern was associated with a progressive increase in fruit‐set across sequential inflorescences in the mesh‐enclosed treatment (Table S2), whereas fruit‐set remained comparatively stable in the shaded and open treatments.

The change in fruit‐set patterns between the mesh‐enclosed and shaded treatments was reflected in fruit production. For all inflorescences, fruit production in the open treatment exceeded that in the shaded and mesh‐enclosed treatments (Figure 2D; Table 1). More generally, plants in the mesh‐enclosed treatment produced relatively few fruits in the earliest inflorescences but continued fruiting more and more across later inflorescences (Figure 2D; Table 1 and Table S2). In both shaded and open treatments, fruit number per inflorescence broadly tracked female flower production (Figure 2), whereas in the mesh‐enclosed treatment, fruit production was more strongly influenced by the marked temporal shift in fruit‐set (Table S3).

### 3.3 Phenotypic Selection in Three Treatments

The opportunity for selection based on relative female fitness was significantly higher in shaded (*I*_shaded_ = 1.30) and mesh‐enclosed treatments (*I*_mesh‐enclosed_ = 1.65) than in open (*I*_open_ = 0.25; shaded vs. open *F*_1,77_ = 11.01, *p* = 0.0014; and mesh‐enclosed vs. open *F*_1,78_ = 12.17, *p* = 0.0008). The selection pattern through female fitness varied among different treatments. In the shaded treatment, a positive selection was evident for the number of female flowers per inflorescence, while in the mesh‐enclosed treatment there was a positive selection on the number of inflorescences (Table 2). In the open treatment, three traits (height, inflorescences, and female flowers per inflorescence) showed significant positive selection, although with relatively lower coefficients (Table 2). The selection pressure for the trait “female flowers per inflorescence” exhibited significant differences among the three treatments (Table 3).

**TABLE 2:** Selection gradient (*β*) via the female reproductive fitness of *Sagittaria trifolia* on five traits (plant height, duration of anthesis, number of inflorescences, male flowers per inflorescence, and female flowers per inflorescence) in three treatments (mesh‐enclosed, shaded, and open). Female fitness was quantified as the total number of seeds produced per plant. Bold font indicates that selection gradients are significantly different from zero.

Gardens	Mesh‐enclosed		Shaded		Open	
Traits	*β*	95% CI	*β*	95% CI	*β*	95% CI
Plant height	0.21	−0.08 ~ 0.59	0.07	−0.40 ~ 0.45	**0.18**	**0.0003 ~ 0.37**
Duration of anthesis	−0.14	−0.47 ~ 0.22	0.11	−0.49 ~ 0.02	−0.03	−0.19 ~ 0.07
No. of inflorescences	**0.38**	**0.03 ~ 0.91**	0.17	**−**0.14 ~ 0.58	**0.18**	**0.07 ~ 0.30**
Male flowers per inflorescence	0.35	−0.02 ~ 0.97	−0.16	−0.64 ~ 0.20	0.11	−0.10 ~ 0.36
Female flowers per inflorescence	0.44	−0.04 ~ 0.80	**1.10**	**0.54 ~ 1.92**	**0.26**	**0.03 ~ 0.46**
**Model fit**	R^2^ = 0.54	*F*_5,29_ = 6.93	R^2^ = 0.70	*F*_5,27_ = 13.99	R^2^ = 0.57	*F*_5,34_ = 11.17

**TABLE 3:** ANCOVA table testing the effects of trait, treatment, and their interactions on reproductive fitness in plants of *Sagittaria trifolia* .

Source	DF	Sum Sq	F value	*p*
Environment	2	7.53	8.25	0.0005
Height	1	35.32	77.38	< 0.0001
Duration of anthesis	1	1.03	2.26	0.14
No. of inflorescences	1	1.99	4.37	0.039
Male flowers per inflorescence	1	8.01	17.55	< 0.0001
Female flowers per inflorescence	1	9.18	20.12	< 0.0001
Height * Environment	2	1.20	1.31	0.27
Duration of anthesis * Environment	2	0.015	0.17	0.98
No. of inflorescences * Environment	2	0.61	0.66	0.52
Male flowers per inflorescence * Environment	2	1.37	1.50	0.23
Female flowers per inflorescence * Environment	2	4.69	25.14	0.0077

## 4 Discussion

### 4.1 Treatment Effects on Reproductive Allocation and Fitness

In the mesh‐enclosed treatment, where access by major flying pollinators was restricted under shaded conditions, *Sagittaria trifolia* generally showed intermediate values for several reproductive traits and, in some cases, tended to maintain anthesis duration and floral production despite reduced light availability. One possible explanation is that reduced effective pollination lowered fruit set and seed development, thereby reducing early reproductive expenditure and allowing plants to continue flowering across successive inflorescences. Such a response would be consistent with the hypothesis that plants experiencing restricted pollination may prolong flowering or maintain floral production, a strategy that has been discussed repeatedly but remains supported by relatively limited empirical evidence (Ashman et al. 2004; Knight et al. 2005; Wesselingh 2007). In the shaded treatment, plants experienced reduced light while retaining access to pollinators, and produced fewer inflorescences and substantially fewer seeds than those in the open treatment, suggesting that reduced resource availability constrained reproductive performance. By contrast, in the open treatment where light was abundant, *S. trifolia* produced significantly more inflorescences and seeds, and showed longer anthesis duration and greater floral display, indicating that higher light availability was associated with enhanced reproductive output. It should be noted that the current study did not strictly quantify lifetime fitness, because *S. trifolia* is a perennial geophyte whose above‐ground shoots senesce after the growing season while underground corms overwinter, making it difficult to track individual genets across years. Nevertheless, a single breeding season with quantification of both fruit production and corm formation can still provide useful information on seasonal reproductive allocation and selection, particularly if interannual resource carry‐over is limited in this species, as suggested by the short persistence of rootstocks.

The similarity in total seed production between mesh‐enclosed and shaded plants suggests that the negative impacts of pollinator loss can be mitigated when plants simultaneously experience resource limitation, given that the two treatments held statistically the same light intensity (Figure S2). This finding supports models predicting that the effects of reduced pollination become less severe under resource constraints (Haig and Westoby 1988; Rosenheim et al. 2014). The likely mechanism involves both compensatory ecological processes and physiological adjustments. Under restricted access of major flying pollinators, some low‐efficiency floral visitors (ants and bugs; Figure S1) may have taken up the vacancy and contributed to pollen transfer, as reported in other systems with generalized pollination (Gómez et al. 1996; Rader et al. 2016). Additionally, extended flowering duration may increase the opportunity for pollen receipt under restricted visitation by increasing the chance of encounters between visitors and flowers. Several species‐specific traits may have facilitated this compensatory response: (1) sequential production of multiple inflorescences enabling rapid adjustment of resource allocation across time; and (2) self‐compatibility allowing selfing when pollinators are of poor mobility and carry pollen over short distances (Horiuchi et al. 2022). Overall, the results imply that under low light, resource limitation may dominate total reproductive output, while restricted pollination can still influence flowering dynamics and allocation across sequential inflorescences of *S. trifolia* .

Our results showed that the percentage of female flowers was stable in all three treatments, suggesting that light and pollinator limitations had no significant effect on sex allocation. This lack of response contrasts with previous studies in species of *Sagittaria* where nutrient gradients (fertilization) or competitive pressures (planting density) more readily induced shifts in gender expression (Dorken and Mitchard 2008; Han et al. 2011), as predicted by the theory of sex allocation (Charnov 1983). It is plausible that the specific nature of our environmental manipulations—altering light and pollinator access, which primarily affect carbon assimilation and mating opportunities—did not trigger the physiological cues (e.g., nitrogen availability) often associated with sex allocation changes in monoecious species (Tonnabel et al. 2017). This finding suggests that, at least with respect to sex allocation, the responses of *S. trifolia* to light and reduced pollination were expressed through changes in whole‐plant reproductive deployment—especially inflorescence production and flowering duration—rather than through shifts in gender expression.

### 4.2 Temporal Dynamics of Resource Allocation Across Sequential Inflorescences

Flower production within individual inflorescences was comparatively more conserved than whole‐plant inflorescence production, but it was not completely invariant across treatments or inflorescence orders. In particular, differences in female and male flower production became evident around the 3rd inflorescence, especially in the shaded treatment. Fruit output reflected the combined effects of female flower production and fruit‐set, the latter of which changed most dramatically in the mesh‐enclosed treatment. The low fruit‐set of the initial inflorescences is consistent with restricted pollination during early flowering. However, its subsequent recovery in later inflorescences suggests either increased activity of alternative pollinators over time or plant reallocation of resources from initially unsuccessful reproductive attempts (Lloyd 1980; Ladio and Aizen 1999). In the open and shaded treatments, fruit‐set remained comparatively stable among inflorescences. Due to higher female flower production, open plants produced more fruits than shaded plants for all three inflorescences.

Plants with sequentially blooming inflorescences appear to be more flexible in allocating reproductive resources. Liu et al. (2012) found that temperature significantly affected flower and seed production in all nine species with multiple inflorescences but not for those with a single inflorescence, suggesting that the flexibility of flower output within an inflorescence is probably more constrained (Zhang et al. 2012). In addition, sequential production of multiple inflorescences also enables more time to buffer and accumulate resources (Yang et al. 2014). While both multiple flowers in a single inflorescence and multiple inflorescences are effective approaches to bet hedging (Brown and McNeil 2006), the latter may suffer less from resource tensions, as the time dimension can allow more resource uptake.

### 4.3 Environment‐Dependent Selection on Reproductive Traits

Our phenotypic selection analysis revealed how environmental conditions alter selective pressures on reproductive traits. Both mesh‐enclosed and shaded treatments showed stronger selection opportunities than the open treatment, implying that under stressful conditions, the difference in performance between individuals was more prominent, which has also been found in a study of 12 orchid species (Trunschke et al. 2017). Notably, the opportunity for selection reflects variance in relative fitness rather than phenotypic trait per se, which might show less variance and more consistent response under stressful treatments (e.g., elongation of leaf petioles under shading, darker pigmentation under high UV; Franklin 2008; Koski and Ashman 2015). Greater opportunity for selection usually indicates stronger phenotypic selection on plant traits (Hou et al. 2024), and consistent with this expectation, we found relatively high selection gradients in the mesh‐enclosed and shaded treatments, especially for the trait of female flower per inflorescence.

The positive selection found in mesh‐enclosed and shaded treatment was on distinct floral traits. Among the mesh‐enclosed plants, inflorescence number was selected to increase reproductive opportunities, which seems an optimal strategy in response to restricted pollination (Rosenheim et al. 2014; Austen et al. 2017). In the shaded treatment, where fruit production was more resource limited than in the open treatment, the expression of more female flowers per inflorescence was favored, probably because of its intrinsic coupling to resource acquisition, as female flowers are more costly (Greenway and Harder 2007). It is interesting to note that plants are selected to achieve higher reproductive success through different routes between these two treatments, considering that they achieved similar female fitness.

In the open treatment, three traits have been detected under selection, favoring genotypes with either more inflorescences, a higher number of female flowers per inflorescence, or taller plants. Interestingly, height was only found to be under selection in the open treatment, probably because in the other two treatments the trait exhibits a stronger response to shading effect, leading height to be decoupled from the resource status of an individual. In our study, selection was not detected for male flowers, probably because the fitness estimate was only for female and there was no observable trade‐off between male and female functions (Table S1).

Several limitations of this study should be noted. Because each experimental treatment was implemented at a single site, treatment and location are confounded. Although the use of identical clonal replicates across treatments controls for genetic variation, residual environmental heterogeneity among sites may also contribute to observed treatment differences; a lack of within‐treatment replication, while common in labour‐intensive selection studies, limits the generality of inferences about treatment‐dependent selection. Additionally, our fitness measure captured only female reproductive success (total seed production), and for a monoecious species such as *S. trifolia* , male function may represent a substantial portion of total fitness; future studies incorporating both male and female fitness components are needed. Furthermore, pollinator restriction (mesh enclosure) was applied only under shaded conditions, so the design does not permit a full factorial separation of light and pollination effects or a formal test of their interaction. Treatment differences should therefore be interpreted as the combined effect of reduced light and restricted pollinator access. Mesh enclosures may also alter microclimate variables such as humidity and ventilation, which we did not measure. Finally, modest sample sizes within treatments restricted our phenotypic selection analyses to linear directional selection gradients. In addition, our experimental plants were derived from multiple natural populations, which broadens the phenotypic range relative to a single‐population sample; this may facilitate detecting selection gradients but could overestimate their magnitude compared to estimates obtained within a single population.

## 5 Conclusions

Our findings demonstrated that whole‐plant reproductive success within a breeding season in *S. trifolia* was influenced by both light availability and pollination service, but that plants can compensate for pollinator loss through plastic resource allocation across sequential inflorescences. The similar final seed yields in mesh‐enclosed and shaded treatments highlight how reproductive compensation can buffer against pollination failure under resource‐limited conditions. These results emphasize the importance of temporal dynamics and a whole‐plant perspective in understanding plant reproductive ecology. They further suggest that species with extended flowering periods and flexible resource strategies (such as perennial *S. trifolia* that can produce multiple inflorescences per season) may be more resilient to pollinator declines than previously assumed.

## Author Contributions

**Hanqing Tang:** formal analysis (lead), investigation (lead), methodology (lead), validation (lead), visualization (lead), writing – original draft (lead), writing – review and editing (supporting). **Can Dai:** conceptualization (lead), data curation (lead), funding acquisition (lead), project administration (lead), resources (lead), supervision (lead), writing – review and editing (lead).

## Funding

This work was supported by the National Natural Science Foundation of China, 32170231 (CD).

## Conflicts of Interest

The authors declare no conflicts of interest.

## Supporting information


**Figure S1:** Background differences of the three common gardens and pollinator diversity of *Sagittaria trifolia* at each garden.
**Figure S2:** The light intensity in the three environments and common flower visitors on plants of Sagittaria trifolia. For light intensity, columns with different lowercase letters are significantly different (F_2,28_ = 14.61, *p* < 0.0001; post hoc comparisons: mesh‐enclosed vs. shaded *p* = 0.95; mesh‐enclosed vs. open *p* < 0.0001; shaded vs. open *p* = 0.0003). Common visitors were Camponotus sp. and Eysarcoris sp. in the mesh‐enclosed environment (left), Episyrphus sp. and Asarkina sp. in the shaded (bottom), and Apis sp. and Eristalis sp. in the open environment (right).
**Table S1:** Phenotypic correlation among flowering traits and fitness of Sagittaria trifolia in three environments. *p*‐values are reported without correction for multiple testing and should be interpreted as exploratory.
**Table S2:** Pearson's correlation between the order of inflorescences and reproductive traits (male and female flowers per inflorescence, fruit‐set, and fruit number) of Sagittaria trifolia in three environments.
**Table S3:** Likelihood‐ratio tests from repeated‐measure mixed‐effects models of female flowers, male flowers, fruits, and fruit‐set across successive inflorescences in Sagittaria trifolia under three environments.

## Data Availability

Data are available via the figshare. link: https://doi.org/10.6084/m9.figshare.27610539.
