# Supplementary material for: Light and Pollination Limitation Alter Patterns of Fitness and Phenotypic Selection in Sagittaria trifolia L.: Insights From Sequential Inflorescences
Source: Ecol Evol. 2026 Jul 1;16(7):e73955. doi: 10.1002/ece3.73955 (PMC13322782; doi:10.1002/ece3.73955)
Supplement: Supplementary file 1 — Figure S1: Background differences of the three common gardens and pollinator diversity of Sagittaria trifolia at each garden. Figure S2: The light intensity in the three environments and common flower visitors on plants of Sagittaria trifolia. For light intensity, columns with different lowercase letters are significantly different (F2,28 = 14.61, p < 0.0001; post hoc comparisons: mesh‐enclosed vs. shaded p = 0.95; mesh‐enclosed vs. open p < 0.0001; shaded vs. open p = 0.0003). Common visitors were Camponotus sp. and Eysarcoris sp. in the mesh‐enclosed environment (left), Episyrphus sp. and Asarkina sp. in the shaded (bottom), and Apis sp. and Eristalis sp. in the open environment (right). Table S1: Phenotypic correlation among flowering traits and fitness of Sagittaria trifolia in three environments. p‐values are reported without correction for multiple testing and should be interpreted as exploratory. Table S2: Pearson's correlation between the order of inflorescences and reproductive traits (male and female flowers per inflorescence, fruit‐set, and fruit number) of Sagittaria trifolia in three environments. Table S3: Likelihood‐ratio tests from repeated‐measure mixed‐effects models of female flowers, male flowers, fruits, and fruit‐set across successive inflorescences in Sagittaria trifolia under three environments. [file ECE3-16-e73955-s001.docx]

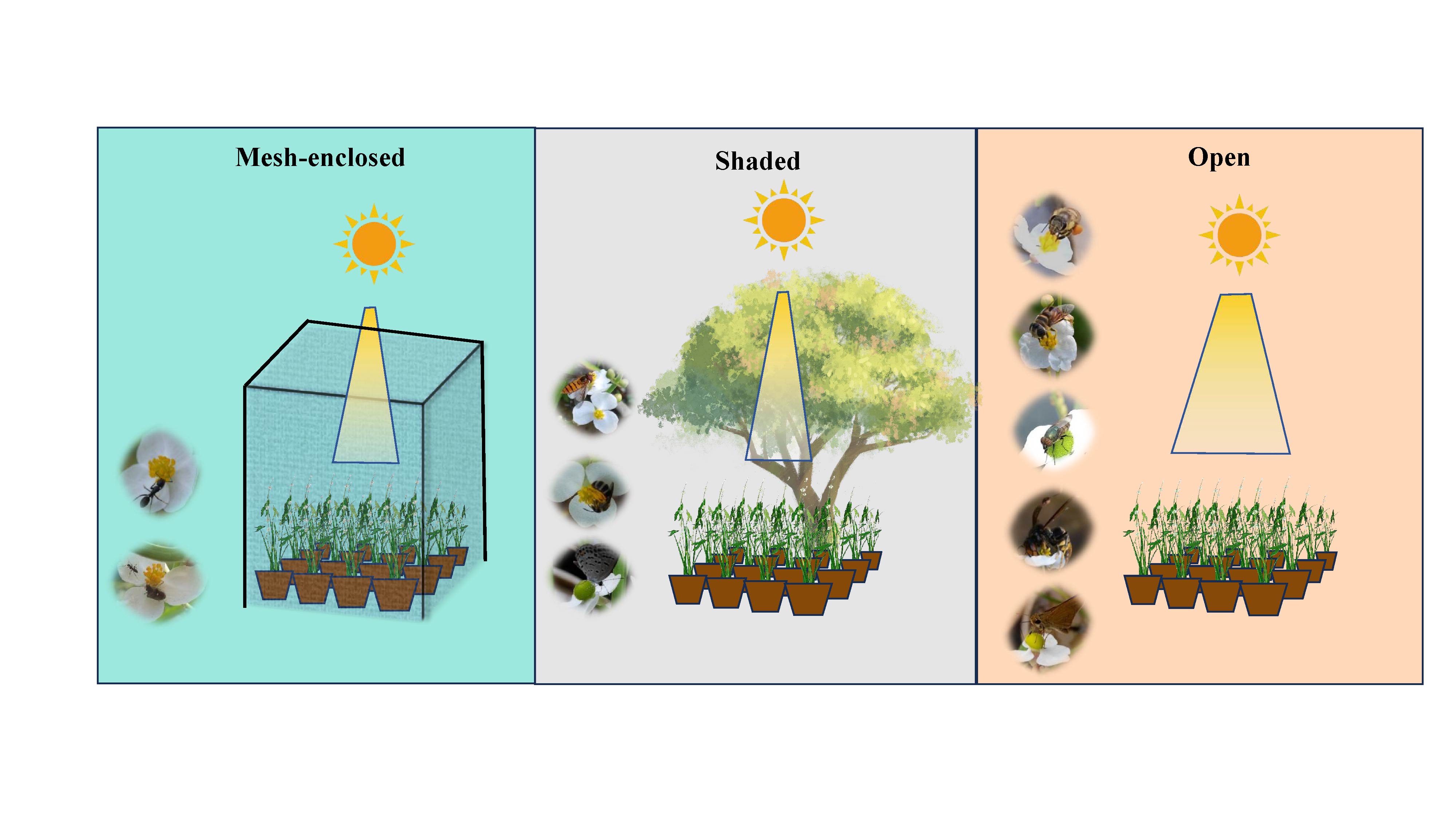


Figure S1 Background differences of the three common gardens and pollinator diversity of *Sagittaria trifolia* at each garden.


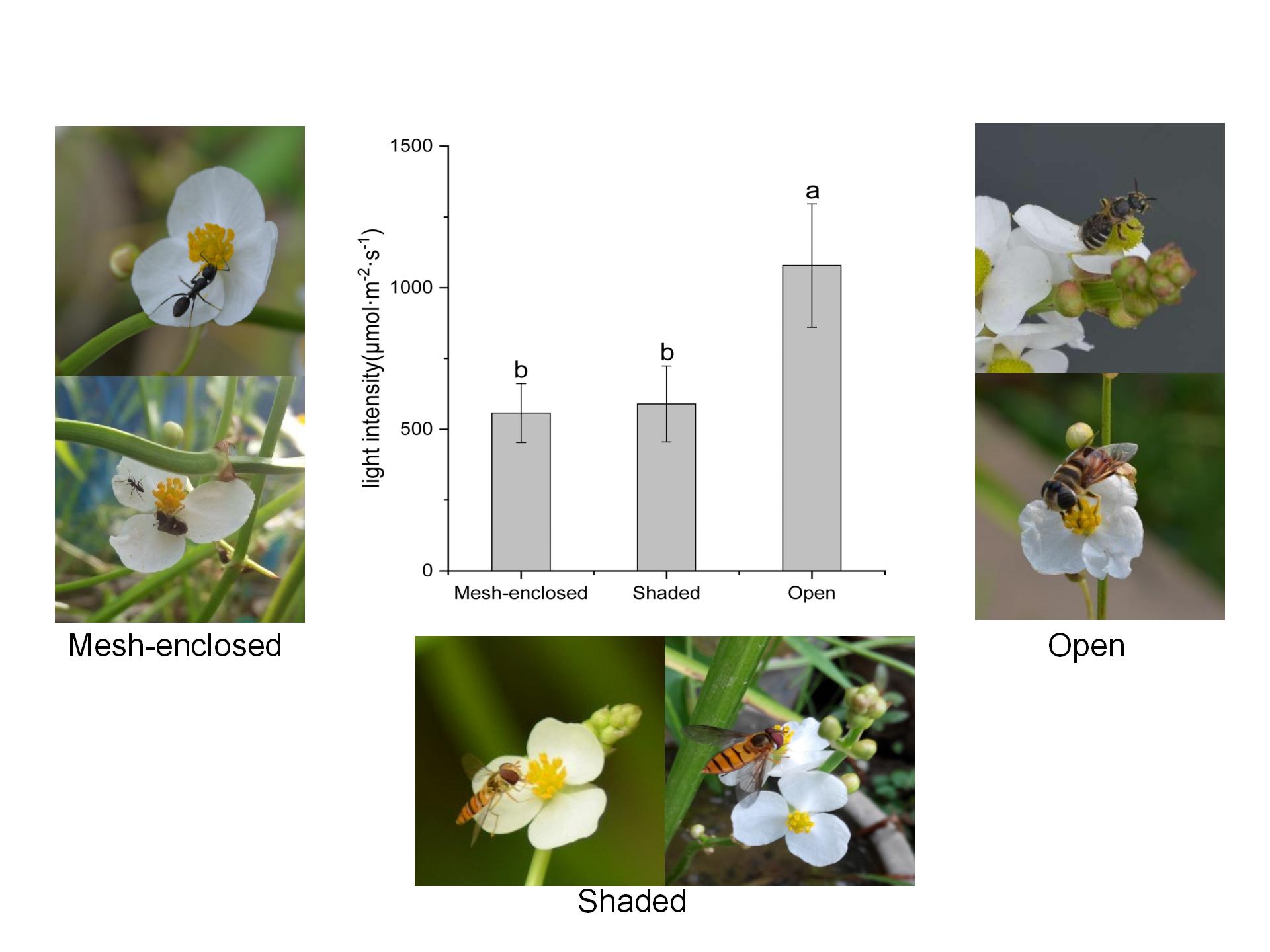


Figure S2. The light intensity in the three environments and common flower visitors on plants of *Sagittaria trifolia*. For light intensity, columns with different lowercase letters are significantly different (*F*_2,28_ = 14.61, *p* < 0.0001; post-hoc comparisons: mesh-enclosed vs shaded *p* = 0.95; mesh-enclosed vs open *p* < 0.0001; shaded vs open *p* = 0.0003). Common visitors were *Camponotus* sp. and *Eysarcoris* sp. in the mesh-enclosed environment (left), *Episyrphus* sp. and *Asarkina* sp. in the shaded (bottom), and *Apis* sp*.* and *Eristalis* sp. in the open environment (right).

|  | Date of first flower | Duration of anthesis | Infloresc-ences | Male flowers | Female flowers | Female percent | Fruit-set | Fruits | Seeds | Corm number |
| --- | --- | --- | --- | --- | --- | --- | --- | --- | --- | --- |
| **Mesh-enclosed** |  |  |  |  |  |  |  |  |  |  |
| Height | 0.22 | -0.21 | -0.03 | 0.33 | 0.40^*^ | 0.06 | 0.40^*^ | 0.52^**^ | 0.45^**^ | 0.05 |
| Date of first flower |  | -0.97^***^ | -0.58^***^ | 0.23 | -0.11 | -0.25 | 0.33 | -0.03 | 0.04 | 0.11 |
| Duration of anthesis |  |  | 0.58^***^ | 0.33 | 0.44^**^ | 0.12 | -0.27 | 0.09 | 0.04 | -0.06 |
| Inflorescences |  |  |  | -0.23 | 0.10 | 0.34^*^ | -0.03 | 0.30 | 0.30 | 0.14 |
| Male flowers |  |  |  |  | 0.51^**^ | -0.58^***^ | 0.39^*^ | 0.47^**^ | 0.50^**^ | -0.07 |
| Female flowers |  |  |  |  |  | 0.35^*^ | 0.34^*^ | 0.69^***^ | 0.62^***^ | -0.14 |
| Female percent |  |  |  |  |  |  | 0.03 | 0.19 | 0.06 | 0.01 |
| Fruit-set |  |  |  |  |  |  |  | 0.59^***^ | 0.41^*^ | 0.11 |
| Fruits |  |  |  |  |  |  |  |  | 0.88^***^ | -0.01 |
| Seeds |  |  |  |  |  |  |  |  |  | 0.005 |
| **Shaded** |  |  |  |  |  |  |  |  |  |  |
| Height | -0.12 | 0.35 | 0.28 | 0.43^*^ | 0.75^***^ | 0.35^*^ | 0.66^***^ | 0.67^***^ | 0.67^***^ | 0.10 |
| Date of first flower |  | -0.83^***^ | -0.30 | -0.09 | 0.08 | 0.12 | -0.13 | -0.19 | -0.14 | -0.27 |
| Duration of anthesis |  |  | 0.46^**^ | 0.43^**^ | 0.35^*^ | -0.21 | 0.28 | 0.32 | 0.25 | 0.30 |
| Inflorescences |  |  |  | 0.02 | 0.12 | 0.003 | 0.20 | 0.45^**^ | 0.27 | 0.30 |
| Male flowers |  |  |  |  | 0.63 ^***^ | -0.36^*^ | 0.35^*^ | 0.47^**^ | 0.44^*^ | -0.03 |
| Female flowers |  |  |  |  |  | 0.43^*^ | 0.74^**^ | 0.82^***^ | 0.81^***^ | -0.07 |
| Female percent |  |  |  |  |  |  | 0.54^**^ | 0.38^*^ | 0.39 | -0.09 |
| Fruit-set |  |  |  |  |  |  |  | 0.77^***^ | 0.76^**^ | 0.12 |
| Fruits |  |  |  |  |  |  |  |  | 0.93^***^ | 0.18 |
| Seeds |  |  |  |  |  |  |  |  |  | 0.18 |
| **Open** |  |  |  |  |  |  |  |  |  |  |
| Height | -0.26 | 0.25 | -0.02 | 0.29 | 0.40^*^ | 0.28 | -0.02 | 0.35^*^ | 0.52^***^ | -0.19 |
| Date of first flower |  | -0.96^***^ | -0.44^**^ | -0.06 | -0.15 | -0.08 | 0.04 | -0.41^**^ | -0.34^*^ | 0.47^**^ |
| Duration of anthesis |  |  | 0.49^**^ | 0.49^**^ | 0.47^**^ | 0.02 | 0.03 | 0.48^**^ | 0.37^*^ | -0.45^**^ |
| Inflorescences |  |  |  | 0.003 | -0.11 | -0.17 | 0.10 | 0.43^**^ | 0.27 | 0.05 |
| Male flowers |  |  |  |  | 0.59^***^ | -0.21 | 0.30 | 0.52^***^ | 0.51^***^ | 0.08 |
| Female flower |  |  |  |  |  | 0.63^***^ | 0.03 | 0.80^***^ | 0.64^***^ | -0.20 |
| Female percent |  |  |  |  |  |  | -0.25 | 0.45^**^ | 0.31 | -0.29 |
| Fruit-set |  |  |  |  |  |  |  | 0.26 | 0.31^*^ | -0.007 |
| Fruits |  |  |  |  |  |  |  |  | 0.79^***^ | -0.19 |
| Seeds |  |  |  |  |  |  |  |  |  | -0.16 |

Table S1. Phenotypic correlation among flowering traits and fitness of *Sagittaria trifolia* in three environments. P-values are reported without correction for multiple testing and should be interpreted as exploratory.

*Note*: * *p* < 0.05, ** *p* < 0.01, and *** *p* < 0.0001.

Table S2. Pearson’s correlation between the order of inflorescences and reproductive traits (male and female flowers per inflorescence, fruit-set, and fruit number) of *Sagittaria trifolia* in three environments.

|  | Mesh-enclosed | Shaded | Open |
| --- | --- | --- | --- |
| Male flower | − 0.024 | − 0.041 | 0.20** |
| Female flower | 0.33*** | 0.14 | 0.42*** |
| Fruit-set | 0.33*** | 0.025 | − 0.053 |
| Fruit | 0.38*** | 0.088 | 0.35*** |

*Note*: ** *p* < 0.01, and *** *p* < 0.0001.

Table S3. Likelihood-ratio tests from repeated-measure mixed-effects models of female flowers, male flowers, fruits, and fruit-set across successive inflorescences in *Sagittaria trifolia* under three environments.

|  | *df* | Chisq | *p* |
| --- | --- | --- | --- |
| **Male flowers** |  |  |  |
| Environment | 2 | 3.15 | 0.21 |
| Inflorescence | 1 | 3.17 | 0.07 |
| Environment* inflorescence | 2 | 2.60 | 0.27 |
| **Female flowers** |  |  |  |
| Environment | 2 | 1.33 | 0.51 |
| Inflorescence | 1 | 58.65 | **< 0.0001** |
| Environment* inflorescence | 2 | 0.97 | 0.61 |
| **Fruits** |  |  |  |
| Environment | 2 | 26.59 | **< 0.0001** |
| Inflorescence | 1 | 27.73 | **< 0.0001** |
| Environment* inflorescence | 2 | 21.24 | **< 0.0001** |
| **Fruit-set** |  |  |  |
| Environment | 2 | 63.13 | **< 0.0001** |
| Inflorescence | 1 | 1.49 | 0.22 |
| Environment* inflorescence | 2 | 16.99 | **0.0002** |
